# UV-A,B,C Emitting Persistent Luminescent Materials

**DOI:** 10.3390/ma16010236

**Published:** 2022-12-27

**Authors:** Suchinder K. Sharma, Jinu James, Shailendra Kumar Gupta, Shamima Hussain

**Affiliations:** 1 Amity School of Physical Sciences, Amity University Punjab, IT City, Sector 82A, Mohali 140306, India; 2 UGC-DAE Consortium for Scientific Research, Kalpakkam Node, Kokilamedu 603104, India

**Keywords:** persistent luminescence, UV-emission, optical properties, phototherapy

## Abstract

The nearly dormant field of persistent luminescence has gained fresh impetus after the discovery of strontium aluminate persistent luminescence phosphor in 1996. Several efforts have been put in to prepare efficient, long decay, persistent luminescent materials which can be used for different applications. The most explored among all are the materials which emit in the visible wavelength region, 400–650 nm, of the electromagnetic spectrum. However, since 2014, the wavelength range is extended further above 650 nm for biological applications due to easily distinguishable signal between luminescent probe and the auto-fluorescence. Recently, UV-emitting persistent materials have gained interest among researchers’ due to their possible application in information storage, phototherapy and photocatalysis. In the present review, we summarize these recent developments on the UV-emitting persistent luminescent materials to motivate young minds working in the field of luminescent materials.

## 1. Introduction

Luminescence or the emission of light is an old phenomenon. The first report of light emission from fireflies and glowworms can be dated back to the period, 1500–1000 B.C., in the Indian holy scriptures “Vedas” and also in Chinese Book of Odes (the Shih Ching). The word for “glowworm” in sanskrit language is “Khadyota” [1]. The reports on the glow of bluish-green color from a stone, by Vincenzo Cascariolo, is another important report in the literature [1,2,3,4]. The stone was named “lapis solaris” (also called bolognian stone or solar stone or sun sponge or spingiasolis) [1,2]. Similar reports can also be found for the glow from decaying fish, fungus and bacteria [1,2,5]. Later, in 1612, La Galla wrote first publication on this first man-made material [1,2]. Thus, the term “phosphor” was coined to distinguish it from the elemental phosphorous [2]. Such afterglow which was discovered and reported in different time domains was later known as “phosphorescence” [2]. In 1888, a German physicist named Eilhard Wiedemann coined the term ‘luminescence’ which included both fluorescence and phosphorescence [2]. The fluorescence and phosphorescence are also termed as short-lived and long-lived luminescence, respectively. The different categories of luminescence were later developed based upon the choice of the different excitation methods [2].

The beginning of modern era can be dated back to the discovery of man-made ZnS:Cu,Co material by Hoogenstraaten and Klasens in the year 1953 [6]. However, the materials could not be commercialized successfully because of its shorter decay time and stability in humid environment. The most intense emission of this material was observed at wavelength 530 nm. In 1971, another important material from the strontium aluminate family, SrAl${}_{2}$O${}_{4}$:Eu${}^{2+}$, was discovered [7]. Matsuzawa et al., in the year 1996, published first article on the persistent luminescence of SrAl${}_{2}$O${}_{4}$:Eu${}^{2+}$,Dy${}^{3+}$ having maximum emission at wavelength 520 nm [8]. Upon searching for the same paper on Google, one can find that the article has already been cited 2347 times (to date). After this report, the aluminate family became popular and was explored in detail by many researchers [9,10,11,12]. The data on the strontium aluminate host family has also been summarized recently by Heggen et al. along with a possible new direction for exploring it further [13]. Among other hosts, Sr${}_{2}$MgSi${}_{2}$O${}_{7}$:Eu${}^{2+}$,Dy${}^{3+}$ from the silicate family was established in the year 2001 by Lin et al. [14]. These materials have found applications in various domains including biology (bioimaging), chemistry (different synthetic procedures), physics (application of materials for decoration, safety signage, and solid state lighting) and material science (engineered materials). The use of these materials in watches and toys can easily be found around us. The common abbreviation used in the literature for persistent luminescence is PersL, as coined in a recent review article by Xu et al. [15]. Hereafter, we will use the same abbreviation (PersL) for persistent luminescence phenomenon. The term PersL material has been used for materials with phosphorescence from minutes to hours. PersL materials are quite similar to an optical battery where the material is first charged for some duration (few s to min), and the emission of light is observed when the material is kept in the dark. Emergency signage used in case of electricity failures, watch dials, decorative objects, toys, for energy storage and others are some of the uses of these materials [15,16].

**Figure 1:**
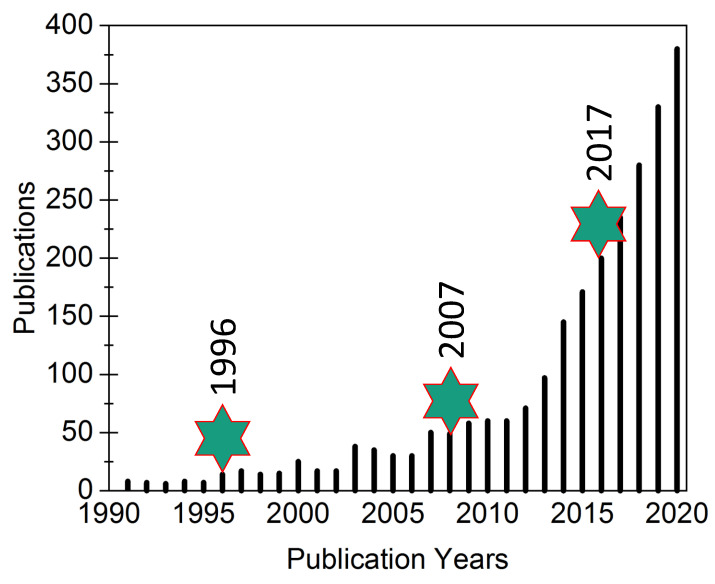
The number of publications on PersL materials as a function of the publication year. Three important years, 1996, 2007 and 2017, are also marked.

As shown in Figure 1, based upon published literature on PersL materials, one can observe three different stages: (a) year 1996 when the first publication by Matsuzawa was published (as discussed in previous section) [8]; (b) year 2007 when another article by Chermont et al. was published where PersL material (silicate host) was used for bio-imaging for the first time [17]; and (c) year 2017 when the first report on the PersL from an organic material was reported [18]. The other articles by Bessiere et al. in 2011 [19] and Maldiney et al. in 2014 [20] are other important publications in the field. Upon looking at the publication year and number of publications/year from the data in Figure 1, one can observe an exponential increase in the number of publications suggesting an increased interest of the scientific community.

## 2. Crucial Parameters for PersL Materials

The type (nature) of defect, their number and energetics within the host band gap are important for a good PersL material. One can engineer new materials by modulating the host bandgap itself or by changing the defect scenario in the host lattice. The PersL materials are expected to possess following important parameters to get required emission color, efficiency and the long lasting luminance:

1. **Trap depth:** The trap (defect) depth within the host lattice is the first and foremost important parameter for a good PersL material. When the trap depth energies are between 0.5–0.6 eV, the material can be effectively charged (trapping) and discharged (detrapping) at room temperature. In fact, instead of intentional defects (0.5–0.6 eV), materials containing unintended defects with trap depth between 1.0–2.0 eV can be utilized for de-trapping under the influence of thermal or optical energy addressed as thermoluminescence (TL) and optically stimulated luminescence (OSL) respectively. The more details on these aspects can be found in Refs. [21,22].
2. **Minimum light output:** PersL is the light output that is observed when an initial excitation is seized. Hence, the two important parameters that prevail after such seizure of excitation energy are: (a) light output, and (b) its duration. Usually in most of the materials, the light intensity decreases by almost 90 % of the initial value in the initial few minutes limiting their commercial aspects. However, a good PersL material is the one in which the duration below which the photopic intensity decreases to an eye perceivable intensity value of 0.32 mcd/m${}^{2}$, exists. This minimum threshold value is important for applications too.
3. **Frequency factor (s):** When the charges are detrapped from defects at room temperature, there exists a competition between re-trapping and detrapping processes leading to the delay in phosphorescence. The charges once trapped are released very slowly from traps at room temperature delaying the overall recombination process at the luminescence center. Due to this competition between the trapping–detrapping–retrapping processes, multi-exponential or hyperbolic decay curve is obtained. The frequency factor (s) is an important parameter and its value depends upon the competition between these different processes. The typical value of ‘s’ is between 10${}^{6}$–10${}^{14}$ s${}^{-1}$ [21]. However, in literature, a value of 10${}^{11}$ s${}^{-1}$ is used, which underestimates the overall phenomenon leading to wrong interpretation.

Overall, the key engineering aspect to prepare new/existing PersL materials is the creation of luminescence centers intentionally having a trap depth between energies, 0.5–0.6 eV, which can be effectively charged and/or discharged at the room temperature.

## 3. PersL Materials: Synthesis

New PersL materials are researched frequently leading to important ideas and innovative applications. In these developments, materials chemistry plays an important role [23]. For example, apart from the preparation method, the influence of crystallinity, particle size distribution and morphology has an impact on the properties and performance of PersL materials. Therefore, the researchers have shown immense interest in nanometre-scale PersL materials. Although there are various methods for the synthesis, the PersL materials with desired spectral and decay time output are challenging. These methods can be classified into two groups:

### 3.1. Conventional Method

**Solid-State synthesis:** The synthesis method is one of the widely used technique to prepare PersL materials. For example, AO- and B${}_{2}$O${}_{3}$-type compounds, which act as precursors, are used to prepare $AB_{2}$O${}_{4}$-type spinel compounds [19,24]. Such materials have shown promising application in Bio-imaging. In this case, the stochiometric amount of precursors are mixed, either manually or using more sophisticated techniques like ball-milling, to thoroughly mix precursors. Such mixture is then heated at high temperatures for prolonged time. For some materials, repeated grinding and calcining steps are followed for even mixing of the compounds and to obtain the final phase of the product (crystallinity). In the case of $AB_{2}$O${}_{4}$-type spinel compounds, temperature as high as 1400 °C is used to obtain the final product. In some reports, the flux is also preferred to lower the calcining temperature of the samples and to obtain highly crystalline final products.

### 3.2. Non-Conventional Methods

Non-conventional techniques for the synthesis of PersL materials are defined as those techniques which do not comprise steps such as continuous grinding and/or heating at elevated temperatures. These techniques are also called wet-chemical routes. Some of these routes are:

#### 3.2.1. Sol-Gel Method

Sol-gel synthesis method comprises of atleast six different steps which includes hydrolysis, polymerization, gelation, drying, dehydration and densification. The product formed in sol-gel method is uniform and possesses better control over the crystallite size, dimensions, shape and morphology of the final product. The method involves both the physical and chemical processes. The process can prepare different oxide-based host matrices with ease. The synthesis of PersL materials, CdSiO${}_{3}$ [25], Sr${}_{2}$SiO${}_{4}$ [26], Y${}_{3}$Al${}_{5}$O${}_{12}$ [27], and LaAlO${}_{3}$ [28], are noticeable contributions in the field.

The method involves primarily two important hydrolysis and condensation reactions and the final output depends on the nature of precursor, solvents, reaction temperature and pH of the sol. In literature, from the synthesis point-of-view, Lu${}_{3}$Al${}_{5-x}$Ga${}_{x}$O${}_{12}$:Ce${}^{3+}$,Cr${}^{3+}$ [29], Gd${}_{3}$Al${}_{5-x}$Ga${}_{x}$O${}_{12}$:Ce${}^{3+}$,Pr${}^{3+}$ [30], CaAl${}_{2}$O${}_{4}$:Yb [31], Sr${}_{2}$MgSi${}_{2}$O${}_{7}$:Eu${}^{3+}$,Dy${}^{3+}$ [32], SrAl${}_{2}$O${}_{4}$, SrSnO${}_{3}$:Pr${}^{3+}$ [33], Y${}_{2}$O${}_{3}$:Dy${}^{3+}$/Nd${}^{3+}$/Sm${}^{3+}$ [34], Li${}_{6}$CaLa${}_{2}$Nb${}_{2}$O${}_{12}$:Eu${}^{3+}$ [35], are important PersL materials synthesized through the sol-gel method. For UV-emitting PersL materials, limited literature based on materials such as Pb${}^{2+}$ ions doped Ca${}_{2}$La${}_{8}$(SiO${}_{2}$)${}_{6}$O${}_{2}$ [36], Lu${}_{2}$SiO${}_{5}$:Pr${}^{3+}$ [37], CaMgSi${}_{2}$O${}_{6}$ [38], could be found.

#### 3.2.2. Combustion Method

Combustion method is another interesting technique to prepare nanoparticles of PersL materials. This technique was developed in late 1990s. In this method, the use of a fuel, either of urea, hydrazine or glycine, is preferred. Fuel plays crucial role in propagation of the reaction via layer by layer heat transfer. The final product in combustion synthesis is mostly powders while some other forms like foam and conglomerates are also reported in the literature. As the reaction in combustion synthesis is highly exothermic, the temperature during such heat release is ∼2500 K. The method is mostly used to prepare oxides and thus require water soluble precursors (mostly nitrates). The method was modified in later stages to use either heated wire, electric spark or laser beams to provide initial temperature for reaction to initiate. Once initiated, the reaction continues on its own due to the presence of fuel.

The final product in this reaction is obtained within 5 min of the initiation of the reaction. Apart from the use of a fuel for reaction, there are two more important parameters that should be fulfilled: (a) the product to be formed should be refractory in nature, and (b) the solution prepared from precursors should be well dispersed and should possess high chemical energy so that the combustion reaction can initiate. The reaction is initiated at temperature ∼500 K in an appropriate atmosphere which is mostly dominant by oxygen gas (usually air) to promote exothermic reaction. Combustion synthesis is characterized by several benefits such as the low reaction duration, low desired initial temperature for reaction initiation, quick synthesis and high final product yield. Moreover, in this method, there is no need for high temperature furnaces as is the case with solid-state synthesis method. Some of the interesting articles in this domain are Refs. [39,40,41].

#### 3.2.3. Hydrothermal Method

The method is another important non-conventional method to prepare nanoparticles of PersL materials. The method is even capable of preparing nanomaterials which are unstable at high temperatures. The solution of either oxides, hydrides, or metal powders is prepared in the suspension form for an initiation of the reaction. The important parameters, temperature (around 573 K) and pressure (around 100 MPa), are controlled to control the shape and size of the final product (nanoparticles). The reaction starts with a nucleation step followed by the growth under controlled conditions of temperature and pressure. All the type of final products, oxides, metal nanoparticles and non-oxides can be prepared using hydrothermal method. The relevant synthesis process to prepare PersL materials can be obtained from Refs. [42,43,44].

#### 3.2.4. Co-Precipitation Method

The co-precipitation is a non-conventional method of preparing PersL materials. The main achievement of this synthesis type is that there is a better control over size of the nanoparticles via control over pH, temperature and concentration of the reactants. The nanoparticles prepared are homogeneous and no agglomeration is obtained for the final product. This method has been applied to synthesize variety of PersL materials, for example Sr${}_{2}$MgSi${}_{3}$O${}_{7}$:Eu${}^{2+}$Dy${}^{3+}$ [45]. The method can also produce nanotues of SrAl${}_{2}$O${}_{4}$:Eu${}^{2+}$Dy${}^{3+}$ [46], and other important hosts as mentioned in Refs. [47,48].

#### 3.2.5. Pechini and Citrate Gel Method

Marcilly and coworkers developed this technique in the year 1970 [49]. The pH during this synthesis is controlled between 6 and 7.5 to dissolve nitrates of precursor salts with citric acid solution. Polybasic chelating agent is changed to a resin and later to transparent gel during synthesis process. The gel is then pyrolized to obtain nanoparticles of the final product. The method is known to prepare multi-component oxides. More recently, the method is modified and is called modified-Pechini method [50,51,52]. In this new modified method, the polymerizable complexes are preferred and added to control the growth kinetics.

The above mentioned conventional and non-conventional synthesis procedures show great potential to prepare nanoparticles with reasonable control of the as-prepared PersL materials [53,54].

## 4. PersL Materials: Wavelength Overview

The most promising application of PersL materials is in bio–imaging as discussed by Maldiney et al. in the year 2014 [20]. PersL materials for such application are red or near-IR emitting around wavelength 700 nm, where there is a very little overlap between emission from the luminescence probe and absorption that of the animal cells, tissue, water and/or melanin. This region is especially important so as to distinguish between the auto-fluorescence from the cells and that of the PersL probe. Moreover, the nanoparticles of such synthetic probes (PersL materials) are generally preferred as they show high carrier mobility in the free-state, enormous specific surface area, as well as exhibit quantum effect. Similarly, visible range emitting PersL materials are preferred for other applications such as signage devices and for other decorative purposes. While most of the discovered PersL materials are emitting within the visible/near-IR wavelength range, very little is published on the UV-emitting PersL materials. The UV-light bands, UV-A (315–400 nm), UV-B (280–315 nm) and UV-C (200–280 nm), play an important role for applications like photo-catalysis, anti-counterfeiting and water-disinfecting, etc. [55]. Thus, recently the attention has been evident focusing on the development of novel UV-emitting photoluminescence (PL) materials [56,57]. However, these PL materials are expected to also emit PersL for some specialized applications.

The rays coming from the sun are another important source of UV-radiations especially that of UV-A and UV-B radiations. The amount of UV radiations received on earth from the sun vary from altitude, weather, season of the year, time of the day and latitude. These UV-radiations cause pigmentation or tanning in humans. UV-A and UV-B induces tanning in the basal cell layer and upper layers of the epidermis, respectively [58,59]. The other after effects of high doses of UV-radiations also include DNA damage and photo-carcinogenesis [60,61,62]. On the positive side, photodynamic therapy (PDT) is another novel technique of curing patients suffering from bacterial infection [58]. Depending upon the choice of UV-radiations for exposure (UV-A/B/C), different phototherapy techniques can be classified [58]. Similarly, there are other application domains (as will be discussed in later sections) for which new and promising UV-emitting materials are desired and require immediate attention of the scientific community.

Almost 95% of the emitted UV-radiations from the sun contain UV-A radiations in the wavelength region 315–400 nm. These radiations are capable of affecting the top layer of skin and can cause premature ageing, wrinkles and some skin cancers. There are two important components to consider while thinking about the existing or new UV-emitting PersL materials: (a) the choice of the host lattice, and/or (b) the choice of the dopant ions. The prominent hosts used by materials scientists to prepare PersL materials are garnets, silicates, phosphates and perovskites, as has been summarized recently by Wang and Mao [63]. On the other hand, the choice of luminescence center, defined as the color emitted by the luminescent materials, is mainly focused on Pb${}^{2+}$, Bi${}^{3+}$, Pr${}^{3+}$, Gd${}^{3+}$, Ce${}^{3+}$ and Tb${}^{3+}$ [63,64].

**Figure 2:**
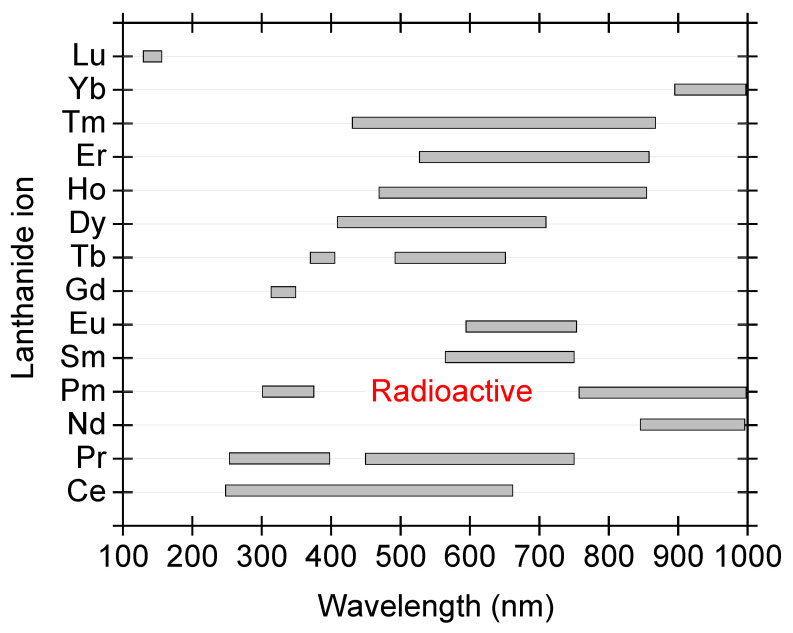
The variation of luminescence emission for all the fourteen lanthanoid ions (Ce to Lu) in different hosts. The marked emission ranges are typical of emission from Ln${}^{3+}$ ions only.

While Pb${}^{2+}$ and Bi${}^{3+}$ are post-transition metals, the other prominent dopants in the literature are rare earth (lanthanoid, Ln) ions. The lanthanoids possess electronic configuration, [Xe]4f${}^{n}$6s${}^{2}$, where the n changes from 1 (for Ce) to 14 (for Lu). Lanthanoid ions can assume the oxidation state of ^2+^, ^3+^ or 4+, with respective loss of 6s${}^{2}$, 6s${}^{2}$ + 4f${}^{1}$ and 6s${}^{2}$ + 4f${}^{2}$ electrons. Then the outside shielding is performed by the 5s${}^{2}$ and 5p${}^{6}$ electrons. They also show contraction effect also known as lanthanoid contraction, which causes a decrease in their atomic (and ionic) radii as the atomic number increases from 1 → 14 [65]. Due to this shielding effect, except Ce${}^{3+}$, other Ln${}^{3+}$ luminescence is not affected much by the choice of the host matrix. The summary of the choice of lanthanoid ion and corresponding emission in different hosts is shown in Figure 2.

Among different 14 lanthanoids, the most promising is Ce${}^{3+}$. Ce${}^{3+}$ with one electron in the 4f orbital is shielded by 5s${}^{2}$ and 5p${}^{6}$ orbitals [66]. The 4f${}^{1}$ state is hardly perturbed by the type of compound it is added in to. However, upon excitation to the 5d orbital, a strong interaction exists, which should be taken care of, while interpreting the luminescence spectra. Depending upon the site symmetry, utmost five distinct 4f → 5d transitions can be observed upon Ce${}^{3+}$ doping. The crystal field leads to a decrease in the lowest of the 5d state by approximately 52,000 cm${}^{-1}$ when measured from the top of 4f levels. This shift is also known as ‘centroid shift’ or ‘barycentre’. The combined effect of spin-orbit interactions and crystal field leads to the redshift (denoted by D) of the first 4f → 5d transitions [66,67,68,69,70,71]. The typical lifetime of Ce${}^{3+}$ 5d–4f transitions varies in the range ∼10–60 ns in different hosts. The Ce${}^{3+}$ PersL can be observed in different hosts, which includes, oxides, sulfides, silicates, garnets, etc. For only Ce${}^{3+}$ doped phosphor hosts, a clear variation in PersL emission maximum from 385 nm to 525 nm can be observed. Among aluminates, SrAl${}_{2}$O${}_{4}$:Ce${}^{3+}$ possesses UV-A PersL emission at 385 nm with afterglow of over 10 h [72,73].

Apart from Ce${}^{3+}$ ion, when we look into Figure 2 to find the promising lanthanoid for an emission in the UV-region (200-400 nm), very limited options are available below 400 nm. The ones with such capability are Ce${}^{3+}$, Pr${}^{3+}$, Pm${}^{3+}$ (also radioactive), Gd${}^{3+}$, and Tb${}^{3+}$. Lu${}^{3+}$ doping is also promising though the emission is in the vacuum ultra-violet region, cf. Figure 2. For all these doping options, the excitation from high energy (for downconversion) is required and thus requires more sophisticated instrumentation (even for the excitation process).

## 5. UV-Emitting PersL Materials

The UV-emitting PersL materials are promising for diverse applications as discussed in previous sections. The literature published on PersL materials emitting in the three different UV domains, UV-A, UV-B, and UV-C, is compiled in Table 1.

From the data, three different rare earth–based dopant ions, Ce${}^{3+}$, Gd${}^{3+}$ and Pr${}^{3+}$, are observed to give PersL emission in the UV-region. Ce${}^{3+}$ doping mostly gives PersL emission in the UV-A region of the electromagnetic spectrum, while the Gd${}^{3+}$ emits mostly in the UV-B region. For Pr${}^{3+}$, the PersL emission is observed in all the three wavelength regions, UV-A, B and C, of the UV-region. PersL emission is based on the excitation of charges to the higher excited state followed by trapping of these charges into the defect states. The charges in these defect states can be detrapped at the room temperature, and then trapped again in same defects, thereby delaying the overall PersL time. When we look into the data in Table 1, undoped SrZrO${}_{3}$ gives the lowest PersL time of 100s with emission in the UV-A region [74], while longest PersL decay time is obtained for LiYGeO${}_{4}$ with emission for 300 h [75] The excitation or charging step can be adopted by either choosing high energy for movement through the conduction band or by using low energy for more localized excitation. Thus, based upon these two aspects, the mechanisms of PersL materials can be divided into the delocalized mechanism [21,22] and the localized mechanism [20]. In the delocalized mechanism, the charge trapping–detrapping occurs via conduction band or valence band for electrons and holes; while in localized mechanism, the trapping–detrapping is similar to that of molecular systems [76].

**Table 1:** Different hosts emitting UV-A, UV-B and UV-C PersL emission. The information for the dopant ions, corresponding decay time and application domain is also provided.

Host	Dopant	Emission λ (nm)	PersL Duration	Application	Reference
**UV-A Emission**					
LiScGeO${}_{4}$	Bi${}^{3+}$	361	>12 h	information storage	[77]
SrLaAlO${}_{4}$	Bi${}^{3+}$	380	60 min	photodynamic therapy	[78]
LiYGeO${}_{4}$	Bi${}^{3+}$	350	72–300 h	biomedical, catalysis	[75]
CaB${}_{2}$O${}_{4}$	Ce${}^{3+}$	365	15 h	UV Phototherapy	[79]
Sr${}_{2}$MgGe${}_{2}$O${}_{7}$	Pb${}^{2+}$	370	>12 h	anti-counterfeiting	[80]
LiScGeO${}_{4}$	Bi${}^{3+}$	365	120 h	photodynamic therapy	[81]
NaLuGeO${}_{4}$	Bi${}^{3+}$	400	63 h	photodynamic therapy	[82]
SrO	Pb${}^{2+}$	390	>1 h	–	[83]
CaO	Pb${}^{2+}$	360	>1 h	–	[83]
MO–Al${}_{2}$O${}_{3}$–SiO${}_{2}$	Ce${}^{3+}$	396	2 min	photocatalysis	[84]
SrZrO${}_{3}$	undoped	395	100 s	information storage	[85]
SrZrO${}_{3}$	Pr${}^{3+}$	300–450	10 min	–	[74]
CdSiO${}_{3}$	Bi${}^{3+}$	360	<5 min	photocatalysis	[25]
CdSiO${}_{3}$	Bi${}^{3+}$	360	<10 min	disinfection	[25]
CdSiO${}_{3}$	Gd${}^{3+}$–Bi${}^{3+}$	344	24 h	photocatalysis	[86]
Zn${}_{2}$SiO${}_{4}$	Ga${}^{3+}$–Bi${}^{3+}$	384, 374	4 h	photocatalysis	[87]
LiLuGeO${}_{4}$	Bi${}^{3+}$–Yb${}^{3+}$	350	15 h	biophotonics	[88]
CaAl${}_{2}$O${}_{4}$	Ce${}^{3+}$	400	>10 h	–	[89]
**UV-B Emission**					
CaZnGeO${}_{6}$	Bi${}^{3+}$	300–600	>12 h	photocatalysis	[57]
CYAS	Pr${}^{3+}$	266/311	>12 h	Germ killing	[90]
Li${}_{2}$CaGeO${}_{3}$	Pr${}^{3+}$	240–330	20min	Sterilization	[91]
MLGB	Bi${}^{3+}$	306	>12 h	multimode imaging	[92]
(Y,Gd)${}_{3}$Ga${}_{5}$O${}_{12}$	Bi${}^{3+}$	313	24 h	optical tagging	[93]
MLGO	Bi${}^{3+}$	310–350	24 h	anticounterfeiting	[94]
LAGO	Pr${}^{3+}$	302	60 h	optical tagging	[95]
(Lu,Y)${}_{3}$(Al,Ga)${}_{5}$O${}_{12}$	Bi${}^{3+}$	302–313	72 h	data encryption	[63]
YGG	Bi${}^{3+}$	316	60 min	–	[96]
YAG	Bi${}^{3+}$	303	60 min	–	[96]
BLAGSO	Pr${}^{3+}$	301	3 h	photocatalysis	[97]
SYSO	Gd${}^{3+}$	299	12 h	dermatology therapy	[98]
**UV-C Emission**					
Cs${}_{2}$NaYF${}_{6}$	Pr${}^{3+}$	250	2 h	sensing/biomedicine	[75]
LaPO${}_{4}$	Pr${}^{3+}$	231	2 h	optoelectronic materials	[99]
SYSO	Pr${}^{3+}$	266	12 h	dermatology therapy	[98]
YPO${}_{4}$	Bi${}^{3+}$	240	2 h	cancer therapy	[100]
Lu${}_{5}$SiO${}_{5}$	Pr${}^{3+}$	200–280	12 h	optical tagging	[37]

## 6. Luminescence Mechanisms

**Figure 3:**
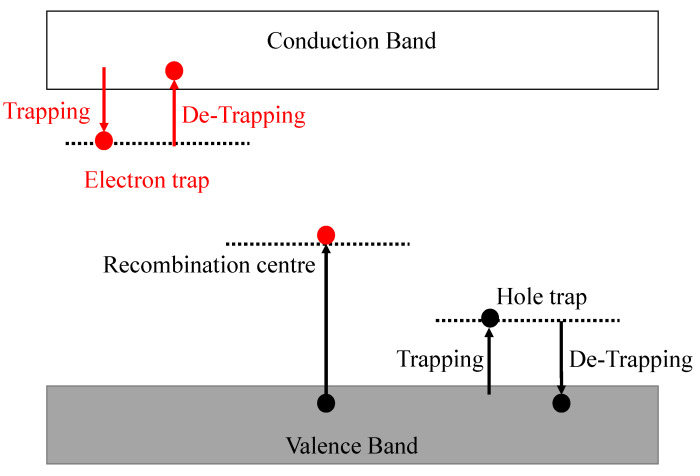
The basic mechanism of charge trapping and detrapping for electron and hole traps. The figure is adapted from Ref. [101].

The mechanism of charge trapping and detrapping is simple, yet complicated. In general, when a sample is excited using an appropriate excitation energy, the electrons move from valence band to the conduction band followed by their trapping at the defect sites. These charges upon trapping require some external stimulation such as heat or optical energy, to get out from such defects. There can be two different ways of charge trapping: (a) one related to electrons (electron trapping), and (b) related to holes (hole trapping), as shown in Figure 3. In both cases, the released charge carriers can recombine with their charge carrier counterpart at luminescence centers producing luminescence due to electron–hole recombination.

### 6.1. Delocalized Mechanism

**Figure 4:**
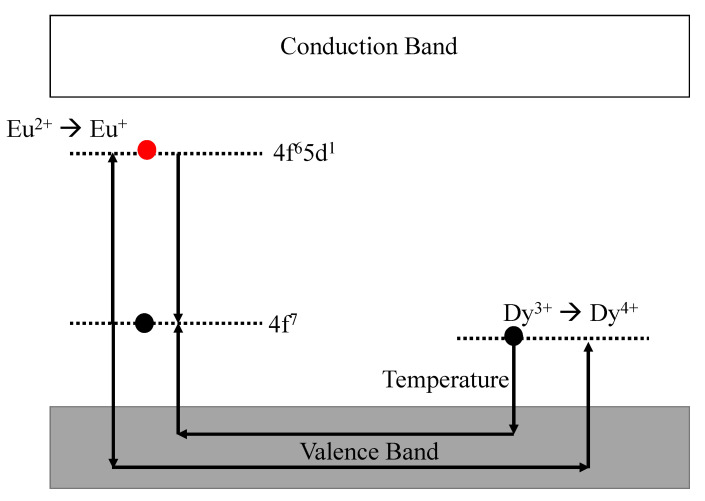
The mechanism of charge trapping and detrapping as proposed by Matsuzawa [8].

Among the most studied mechanisms of charge recombination (which produces luminescence) is delocalized mechanism. Matsuzawa was the first person to explain the mechanism in *M*Al${}_{2}$O${}_{2}$:Eu${}^{2+}$ (*M* = Ca and Sr) material, as shown in Figure 4, assuming holes as the main charge carrier determined using photoconductivity studies [8]. The holes (or traps) are considered to be due to Sr${}^{2+}$ vacancies. When the incident photons excite Eu${}^{2+}$ ions, Eu${}^{+}$ is formed due to escape of a hole. This hole is captured by Dy${}^{3+}$ converting to Dy${}^{4+}$. The thermal energy due to room temperature is considered to be sufficient for these holes to detrap back to the valence band. This follows trapping of these hole back at Eu${}^{+}$ converting to Eu${}^{2+}$ again due to electron–hole recombination at room temperature producing PersL.

After the initial work of Matsuzawa, further work in this direction was perfomed by Aitasalo in the year 2003 [102]. In Aitasalo’s mechanism, the electrons are directly trapped at defects and the holes are trapped at calcium vacancies. Matsuzawa model was rejected by Aitasalo as the PersL from the non-Dy${}^{3+}$ doped sample could not be explained by Matsuzawa. Later in the year 2005, Dorenbos and Clabau proposed two different models of charge trapping–detrapping followed by their recombination at room temperature [103,104]. In the explanation from Dorenbos, the higher states of Eu${}^{2+}$ are determined to be within conduction band. This implies that upon excitation the electrons are within conduction band changing Dy${}^{3+}$ to Dy${}^{2+}$ with a trap depth of 0.9 eV in both the cases. Upon increasing the temperature, the electrons stored in these 0.9 eV traps come out and recombine at the luminescent center. On the other hand, Clabau determined that the electron spin resonance (ESR) signal due to Eu${}^{2+}$ ions decreases as a function of excitation energy suggesting a pathway involving Eu${}^{2+}$ ions. Upon detrapping, the concentration of Eu${}^{2+}$ was found to increase further inferring that the trapping might be at the Eu${}^{2+}$ excited state. This situation (interpretation) contradicts models by Dorenbos and Aitasalo. However, in reality, in contrast to Dorenbos, based on the temperature dependent photoconductivity measurements, no direct migration of electrons through conduction band and the nature of traps upon Dy${}^{3+}$ doping, could be observed [105]. Both Dorenbos and Clabau used electron trapping model, while vacancies were considered to be due to missing oxygen in the lattice.

### 6.2. Localized Mechanism

**Figure 5:**
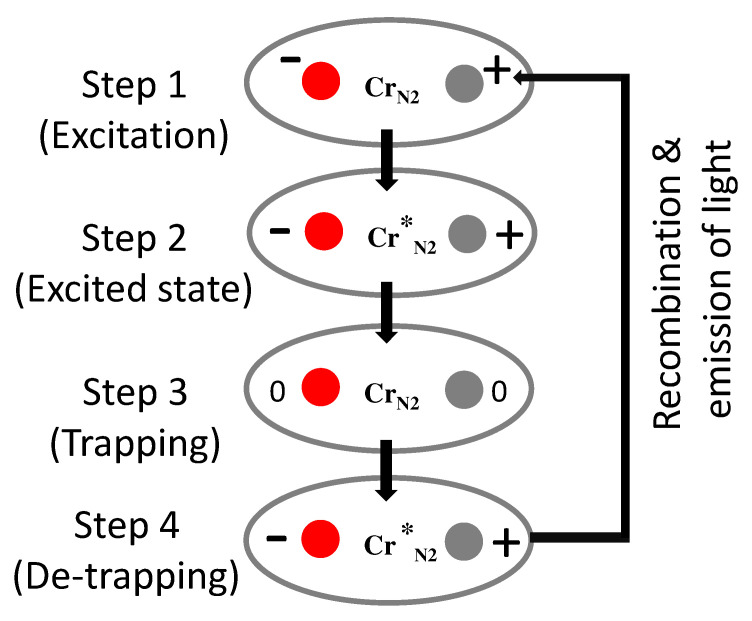
The localized mechanism of charge trapping and detrapping followed by recombination which produces near-IR light emission. The figure is adapted from Ref. [19].

In the localized mechanism, the origin of PersL is the presence of neighboring antisite defects which are close to the luminescent centre. The same can be explained using an example of Cr${}^{3+}$ substituting Ga${}^{3+}$ ion in spinel ZnGa${}_{2}$O${}_{4}$–matrix host. Antisite defects are the defects resulting from the exchange in site positions of *A* and *B* ions in the spinel structure $AB_{2}$O${}_{4}$ [24,76,106,107,108,109]. An important point in this mechanism is that the luminescent centre liberates an electron-hole pair during excitation without changing its oxidation state [76]. The steps that are followed for charge trapping detrapping and recombination in such type of mechanism, for example in ZnGa${}_{2}$O${}_{4}$:Cr${}^{3+}$, is shown in Figure 5. The steps followed are:

- **Step 1:** the excitation of the Cr${}_{N2}$ ions.
- **Step 2:** the excitation is dissociated by the local electric field into an electron and a hole.
- **Step 3:** The excitation is thus trapped in the vicinity of Cr${}^{3+}$ in the form of a pair of neutral defects, while Cr${}^{3+}$ returns to its ${}^{4}$A${}_{2}$ ground state. Electrons and hole can then migrate far from Cr${}^{3+}$ ion, so that this storage mechanism can proceed many times with the same Cr${}_{N2}$ ion.
- **Step 4:** the reverse reaction (electron–hole release and capture by Cr${}^{3+}$) is thermally activated followed by recombination or release of photons.

Such mechanism does not require movement of charges through the conduction band and is known as the localized mechanism. More details of this type of mechanism have been discussed in Refs. [19,20,76].

### 6.3. New Mechanism by Dorenbos

Usually while discussing mechanism of PersL materials, electron trapping and release is preferred and discussed. More recently, in 2018, Dorenbos proposed another mechanism based on the hole-trapping model. Before this, the hole-trapping mechanism was used in the field of semiconductors, and was hardly used in the field of wide band gap inorganic compounds [110]. Such mechanism deals with the difficulties that arise from hole trapping, charge transfer luminescence and luminescence quenching of Eu${}^{3+}$ emission. In this mechanism, the hole ground state of a trivalent lanthanoid is placed at the same location as the electron ground state of the corresponding divalent lanthanoids. Quenching by hole ionization to the valence band then appears a mirror image to quenching by electron ionization to the conduction band. The excited hole state is given by the upside-down Dieke diagrams, and the quenching is described by upside-down configuration coordinate diagrams. The reader is referred to Ref. [110] for further reading.

## 7. Future Direction

Despite several attempts to prepare and understand the UV-emitting PersL materials, the mechanism remains unclear and deserves due attention of the scientific community. The most controversial of all is Strontium Aluminate phosphor, whose mechanism has been under debate for the past 25 years now. Looking in to the role of the trapping centers for applications such as information storage and optical tagging, it is very important to elucidate the mechanism of charge storage, their release and re-trapping at room temperature and even at high temperatures. For example, in the case of Ce${}^{3+}$ doped materials, the lowest of 5d levels (5d${}_{1}$) should be very close to the conduction band for efficient trapping and detrapping at the room temperature. Apart from physical efforts to synthesize new materials, more smart ways like preparing vacuum referred binding energy (VRBE) diagrams should be preferred before actual synthesis of the phosphors [111].

From the prior knowledge of host bandgap and corresponding energetic excitation that causes a maximum photocurrent upon Ce${}^{3+}$ doping, the information about lowest T${}_{2g}$ (for cubic environment) can be determined before the synthesis step. Based on these procedures, afterglow emission change from 2 min to maximum of 1200 min has been reported. Similarly, the duration of the PersL decay time depends upon the delocalization of Ce${}^{3+}$ 5d electrons and their separation from the top of the conduction band. Co-doping such phosphors with other suitable co-dopant ions (e.g., Mn${}^{2+}$, Eu${}^{2+}$/${}^{3+}$, Tb${}^{3+}$) increases the PersL decay time and some new series of materials using energy-transfer phenomenon, can be prepared.

Overall, several compounds with excellent PersL decay emission have already been reported. However, the challenges like particles with small grain size, desired morphology, and emission window exhibiting higher efficiency, are still open. It is known that the PersL decreases with decreasing grain size of particles. However, if it is true for all type of hosts (organic and/or inorganic) or for only few, is still unknown. The pathways to solve such issues require immediate attention of the scientific community. Another area of immense interest for researchers’ working on PersL, is to prepare good, efficient phosphors for security and surveillance purposes.

## 8. Conclusions

The recent developments on PersL materials especially those emitting UV radiation have found applications in photodynamic therapy, information storage, anticounterfeiting, photocatalysis, etc. To prepare UV-emitting materials, which can be divided into UV-A, UV-B and UV-C regions, one needs to follow smart techniques (calculations) rather than physical efforts. While VRBE gives us information about the exact location of rare earth ions in individual hosts, the options to prepare UV-emitting materials is limited as the transitions in lanthanoids are restricted because of their f–f nature (for Ln${}^{3+}$ ions). However, if one considers the Ln${}^{2+}$ ions, and an f–d transition, the emission can vary quite a lot. Only few of the rare earth ions, Gd${}^{3+}$, Ce${}^{3+}$ and Pr${}^{3+}$, are capable of emitting in the UV-region. The Gd${}^{3+}$ and Pr${}^{3+}$ emission is independent of the host crystal field due to shielding of the outermost electrons. The most promising among all is Ce${}^{3+}$ whose 5d shell is affected by the choice of host lattice and the emission can be tuned from UV to red region. Herein, we have summarized such materials and found it to be promising to work on these materials emitting UV–PersL.

## Data Availability

All data are provided within this manuscript.

## Abbreviations

The following abbreviations are used in this manuscript:

PersL	Persistent Luminescence
PL	Photoluminescence
TL	Thermoluminescence
OSL	Optically Stimulated luminescence
VRBE	Vacuum referred binding energy
